# Comparison of Two *Leptospira* Type Strains of Serovar Grippotyphosa in Microscopic Agglutination Test (MAT) Diagnostics for the Detection of Infections with Leptospires in Horses, Dogs and Pigs

**DOI:** 10.3390/vetsci9090464

**Published:** 2022-08-29

**Authors:** Katrin Strutzberg-Minder, Astrid Ullerich, Karen Dohmann, Jan Boehmer, Marga Goris

**Affiliations:** 1 IVD Innovative Veterinary Diagnostics (IVD GmbH), 30926 Seelze, Germany; 2 OIE and National Collaborating Centre for Reference and Research on Leptospirosis, Department of Medical Microbiology and Infection Prevention, Amsterdam University Medical Centre, 1105 AZ Amsterdam, The Netherlands

**Keywords:** *Leptospira*, leptospires, serovar Grippotyphosa, MAT, horse, dog, pig

## Abstract

**Simple Summary:**

Leptospires are bacteria of major health concern as they can cause severe systemic diseases in humans and animals alike. In routine diagnostics, the detection of a leptospirosis heavily relies on the analysis of specific antibodies that were generated by the immune system of an infected species. The most widely used test for the detection of such antibodies is the microscopic agglutination test which itself depends on defined types of laboratory grown Leptospires, so-called serovars. A good judgment of the behaviour of these serovars is vital for interpreting test results and clearly defining what type of Leptospires the patient is infected with as this may have implications for therapy and prognosis. In the following, a study on the reaction pattern of a certain type of serovars named ‘Grippotyphosa’ that itself can be divided into two differently reacting subtypes was conducted. It turned out that, depending on the animal species sampled, a differing reaction patterns to the two Grippotyphosa subtypes in the diagnostic test reflected different distribution of these subtypes in the respective populations. In the future, these insights will further improve test performance and assessment or results.

**Abstract:**

The MAT test is of great importance in the diagnosis of leptospiral infections. Based on various differences, the serovar Grippotyphosa has been divided into two types, Moskva V and Duyster. Differences or similarities of the two type strains in the context of leptospiral diagnostics have not yet been elucidated in more detail; therefore both strains were analysed in MAT diagnostics for the detection of leptospiral infections in pigs, dogs and horses. Serum samples from 2996 pigs, 55 dogs and 35 horses, as well as vitreous and/or aqueous fluid samples from these and 13 additional horses were analysed by MAT; available supplementary samples were tested for leptospires by PCR. In pigs, 92.6% of the samples with both strains received an identical titre result in the MAT test, whereas in dogs and horses only 53.0% and 43.6% had concordant results. Since infections with the serovar Grippotyphosa occur more frequently in dogs and horses overall, more differences were observed here. In the case of discrepant serological results, supplementary samples and PCR examinations were not able to add information on the true status. Further analyses of follow-up studies or at least serum pairs from dogs and horses infected with the serovar Grippotyphosa are necessary.

## 1. Introduction

The MAT using live antigens is the most widely used serological test to check for anti-leptospiral antibodies, and it is still the reference test against which all other serological tests are evaluated, although the latter is now subject to critical review [1]. The MAT is the recommended method for the detection of immune response caused by leptospirosis for the following purposes: individual animal freedom of infection and for surveillances to determine the prevalence of *Leptospira* infection [2]. For optimum sensitivity, it should use antigens representative of all the serogroups known to exist in the region in which the animals are found and, preferably, strains representing all the known serogroups [2]. According to the OIE-Manual, the sensitivity of the test can be improved by using local isolates rather than reference strains, but reference strains assist in the interpretation of results between laboratories. While there are other methods such as lateral flow methods and ELISA to detect infection with specific serogroups or leptospires in general, the quality and significance of the results always depend on the antigens and conjugates used in the tests. The MAT is very valuable and flexible to use as it allows the detection of antibodies in virtually all body fluids of all animal species without major methodological adjustments, although the MAT method is more complex to perform as cultures of leptospires must be kept on hand for all serovars or serogroups to be tested [1]. Furthermore, the MAT not only gives a qualitative answer, but also allows an assessment of the infection phase by titre level and often gives an indication for the serogroup causing the infection [3], especially if serum pairs are available. The latter is not possible with lateral flow tests and most ELISA’s, nor is it so easy even with molecular biological methods. The molecular biological identification of the serogroup so far also required cultures of leptospires [4], which do not have an appropriate cost–benefit ratio in diagnostics and of course need a lot of time according to the growth characteristics of leptospires. Although pathogenic leptospires can now routinely be detected directly by real-time PCR [5,6,7,8,9,10], additional information from the detection of specific antibodies against leptospires by MAT is very helpful because it: (I) can confirm the infection with leptospires, (II) can detect infections when the pathogen content in available samples is below the detection limit of PCR in some phases of infection, especially because of the biphasic nature of leptospirosis, and (III) often allows identification of the presumed causative serogroup and can thus provide general epidemiological information on circulating serogroups [1,11,12]. Recent studies have again impressively demonstrated the specificity of MAT, although an IgM ELISA showed a slightly better sensitivity in human diagnostics [13]. Nevertheless, the low sensitivities of both tests in the early acute phase are consistent with the dynamics of the humoral immune response. In fact, the diagnostic roles of direct and indirect approaches are highly interdependent. Approaches for direct detection are generally more accurate in early infection, while indirect serologic approaches have greater sensitivity later in infection [1]. However, there is no perfect diagnostic test, and every test has its advantages and disadvantages, so that different tests are particularly suitable or unsuitable for different diagnostic purposes. Therefore, the diagnosis of leptospirosis should be multifaceted and not based on a single test but should consider many factors: possible exposure, clinical presentation and laboratory values, as well as the results of several direct and indirect diagnostic tests [1], MAT being one of them [14,15].

Small mammals (esp. rodents) are the most important natural reservoir of leptospires worldwide. During outbreaks of leptospirosis in field workers in Germany, *Leptospira* kirschneri serovar Grippotyphosa was detected in field mice associated with the human cases in 2007 and 2014 [16,17]. Based on these results, it can be assumed that field mice or field hamsters are also the natural reservoir for serovar Grippotyphosa for animals in Germany. Transmission of leptospires usually occurs through direct or indirect contact (contaminated water, mud) with the urine of infected carrier animals, which can excrete the pathogen in high numbers even without being sick themselves. Leptospires usually enter the organism through small skin injuries and through the mucous membranes of the eye, nose and mouth.

A surveillance study in Germany showed that within the period from 2011 to 2016, antibodies against serovar Grippoyphosa could only be detected in 0.8% of all swine serum samples tested [18]. Studies from France and Italy showed that even in these countries, antibodies against Grippotyphosa are rarely found in pigs [19,20].

Antibodies against serovars Grippotyphosa and Sejroe were most frequently detected in dogs in southern Germany (1990–2004; [21]) and serovars Australis and Copenhageni in northern Germany (2003–2006; [22]); vaccinations were not considered.

In a recent study of dogs from Berlin, antibodies against the serovars Australis (24%), followed by Grippotyphosa (20%) and Pomona (9%) were the most common serovars [23]. Further, more recent studies from Switzerland [24], Spain [25] and Italy [26] have shown that antibodies against serovar Grippotyphosa, with varying frequency from 4.5% to 7.2% in Europe, is nevertheless important in dogs.

Seroprevalence and isolation studies indicate that the horse is susceptible to a wide range of incidental infections. As with all other animal species infected with leptospires, most infections in horses are subclinical, but severe diseases also occur [27]. While serovars belonging to the Pomona and Grippotyphosa serogroups are the major associated serovars with disease (abortion, stillbirth, sick foals and equine recurrent uveitis (ERU)) in horse in USA [28], serovar Grippotyphosa is commonly associated with ERU in Europe [29]. In sera from 314 horses examined between May 2012 and November 2013, antibodies against serovar Icterohaemorrhagiae were found in 11.1%, followed by Bratislava at 9.6% and Grippotyphosa at 1.9% in Middle Germany [30]. As the detection of antibodies in the serum of horses does not correlate with ERU, vitreous fluids (VF) are tested for direct and indirect antibodies to confirm the diagnosis before vitrectomy [31,32]. Even though a lateral flow test based on LipL32 for the detection of antibodies in intraocular samples has been evaluated in meantime [33], an examination of intraocular samples by MAT, as already described above, also allows a semi-quantitative analysis in the form of titres and a possible indication of the infecting *Leptospira* serogroup [15]. In a study of VF from 225 horses with ERU, antibodies against leptospires were detected in 35.1% of the cases, among which MAT reactions with serovar Grippotyphosa were the most frequent (84.8% of the positives), followed by Pomona (22.8%) and Icterohaemorrhagiae (10.1%; [34]). Recent studies show that Grippotyphosa is dominant in both serum and VF samples from horses in Germany, which could be confirmed by cultural evidence [29,35]. Furthermore, it could be shown that antibody titres of the serovar Grippotyphosa correlated with disease severity in ERU [36].

Based on various differences described by Wolff and Bohlander (1952) [37], Steinen et al. (1992) [38] and Hartskeerl et al. (2004) [29], the serovar Grippotyphosa has been divided into two types, i.e., Grippotyphosa type Moskva V (Moskva) and Grippotyphosa type Duyster [29]. This division in types has been recognized by the Subcommittee on the taxonomy of *Leptospiraceae* in the meeting of 18 September 2007 in Quito [39]. Because of apparent phenotypic and genotypic differences between the strains Moskva and Duyster found in a previous study [29], we wanted to compare the two type strains in routine diagnostic MAT testing. To our knowledge, a comparison of these two strains when used in MAT in diagnostics has not yet been investigated or published. The aim of the study was to analyse the impact of different type strains of the same *Leptospira* serovar on the diagnostic result of the MAT for different animal species.

## 2. Materials and Methods

### 2.1. Samples

All samples for testing were obtained in connection with diagnostic investigations within the period 1 August 2020 to 31 December 2020. Serum samples (BS) from 2996 pigs, most of them with history of reproductive problems in sows, were analysed by MAT. Serum samples (BS) from 55 dogs (8 male, 10 females, 37 no information on gender; age ranged from months to 14 years, but there was no information about the age for 23 dogs) were analysed by MAT. Paired urine samples were also available from 23 of the 55 dogs and were tested for pathogenic *Leptospira* by PCR. Vitreous fluid (VF) and/or aqueous fluid (AF) from 48 horses (21 male/27 female; age ranged from 2 to 17 years or no information about the age in 10 cases) and paired blood serum (BS) samples from 35 of these horses from Germany and neighbouring European countries, in total 115 samples, were analysed by MAT. VF and AF samples were also tested for pathogenic *Leptospira* by PCR.

### 2.2. MAT

The MAT was carried out according to the instructions of the OIE Terrestrial Manual 2018. All samples were routinely tested against a set of strains from different serogroups (see Table 1). For this question, mainly the results obtained in parallel with the leptospiral strains of the serovars Grippotyphosa type Moskva V (shortly: Moskva; KIT code: KIT0145) and type Duyster (shortly: Duyster; KIT code: KIT0214), both species *Leptospira* kirschneri, were compared, and further results with other strains of other serovars or PCR results were only considered for further evaluation of the two comparison results. Both Grippotyphosa type strains and all others were obtained from the Leptospirosis Reference Centre (Amsterdam UMC, University Medical Centers, Amsterdam, The Netherlands). If sufficient sample material was available, all samples with qualitatively different results were repeatedly examined in the MAT (see Tables S4–S6).

In dogs and horses, reciprocal end point titres < 25 (negative), 25, 50, 100, 200, 400, 800, 1600, 3200 and >3200 were reported and transferred to titre levels and log-titres for further analysis (Table 2a). In the context of herd diagnosis in pigs and considering the compulsory reporting of leptospirosis in pigs in Germany, reciprocal titres of <100 (negative), 100 to 3200 and >3200 were found in accordance with the widely accepted minimum significant titre of 1:100 as significant for an infection ([40]; Table 2b).

### 2.3. Detection of Pathogenic Leptospira by PCR

Nucleic acid was prepared from 300 µL of VF, AF or urine samples using the MagMAX™ Pathogen RNA/DNA kit and the MagMAX™ Express processor (ThermoFisher Scientific, Wesel, Germany) according the protocol of the manufacturer. The PCR testing for pathogenic *Leptospira* based on the method of Ferreira et al. (2014) [7].

### 2.4. Statistical Analyses

**Figure 1:**
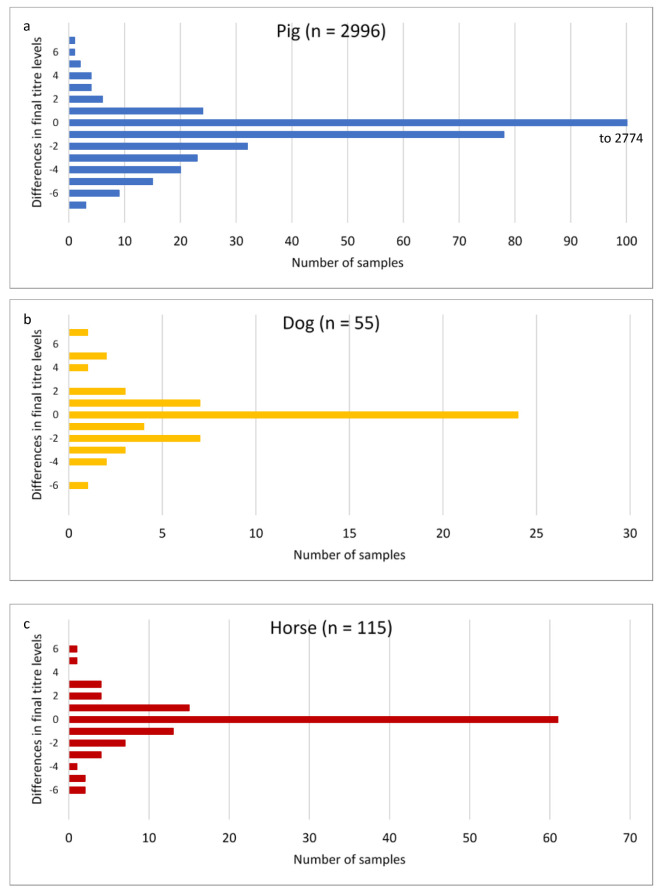
Number of samples with differences in final titre levels of MAT between Grippotyphosa type strains Moskva and Duyster. For all analysed samples from pigs (**a**), dogs (**b**), and horses (**c**). Differences <0 indicate higher titre levels with Duyster; differences >0 indicate higher titre levels with Moskva; 0 indicates no differences in titre levels between the two strains. In order to show the distribution of the differences and the different number of samples more clearly, the *x*-axis was adjusted differently for the different animal species.

The statistical evaluation was descriptive. Since the result values (titre as reciprocal value of dilution and measure for detectable antibodies against leptospires) of the MAT are rank values, the titres were also logarithmised in some analyses for better comparability (Table 2). The deviations or the agreement of the titre levels determined by MAT between the different type strains were first analysed by forming differences (Figure 1 and Table S1).

**Figure 2:**
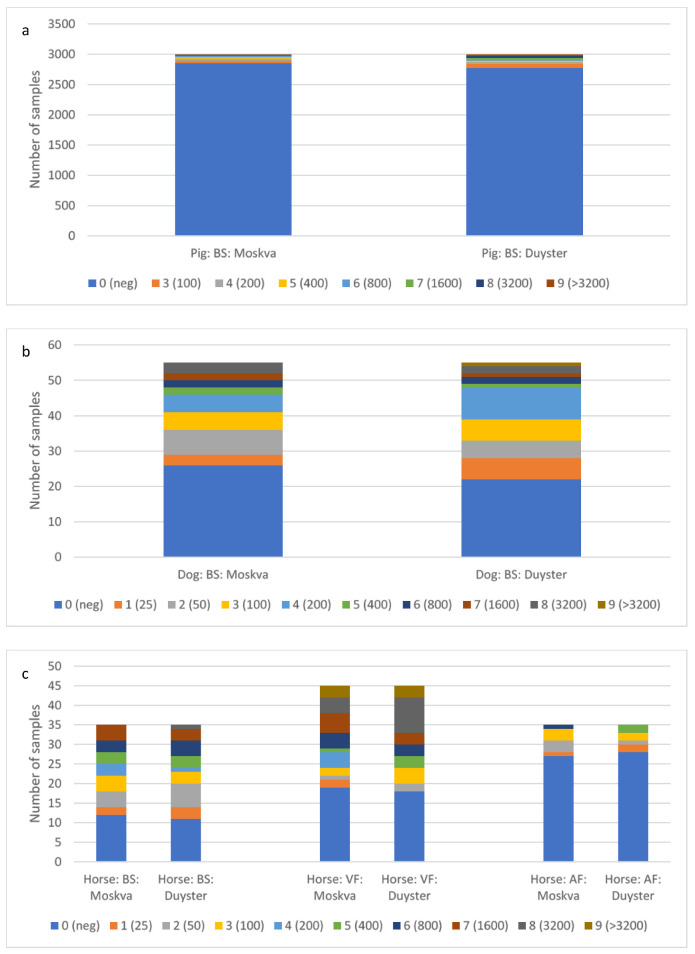
Frequency of MAT titre levels by animal species: (**a**) pig, (**b**) dog; (**c**) horse; and sample material per Grippotyphosa type strain (Moskva and Duyster). BS: Blood serum; VF: vitreous fluid; AF: aqueous fluid; 0–9: titre levels, titres in parentheses; neg: negative; see Table 2.

**Figure 3:**
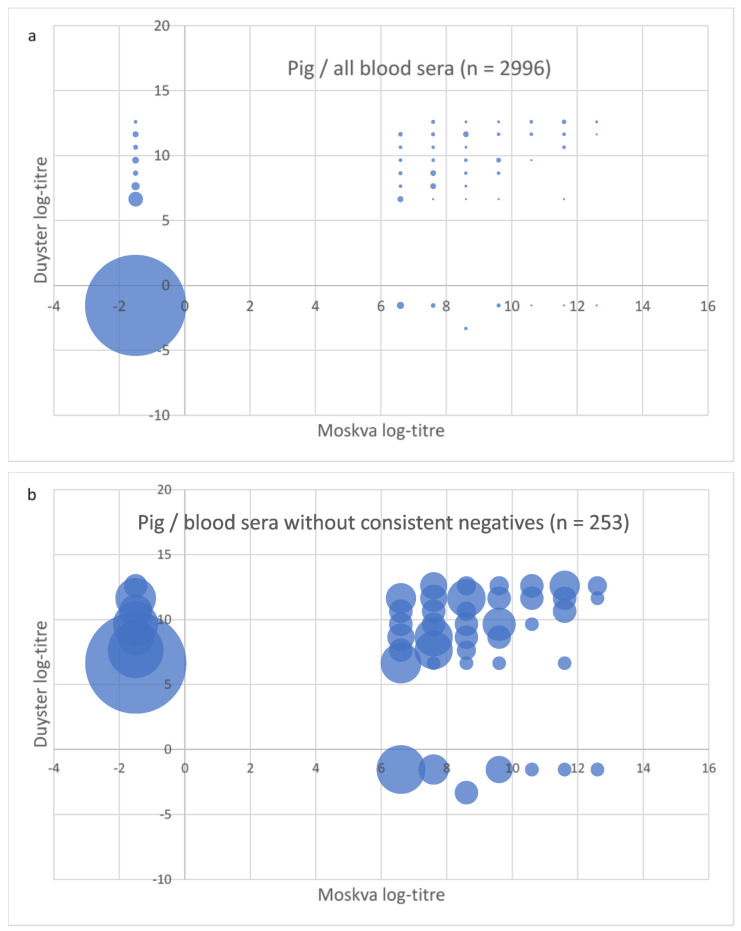
Plot of MAT test result pairs with both Grippotyphosa type strains Moskva (*x*-axis) and Duyster (*y*-axis) for the samples from pig: (**a**) for all serum samples; (**b**) for better illustration without the consistent negative samples (n = 2743), as these accounted for a percentage of 91.6%. The log2 values of the MAT titres (log-titre) are shown as indicated in Table 1.

**Figure 4:**
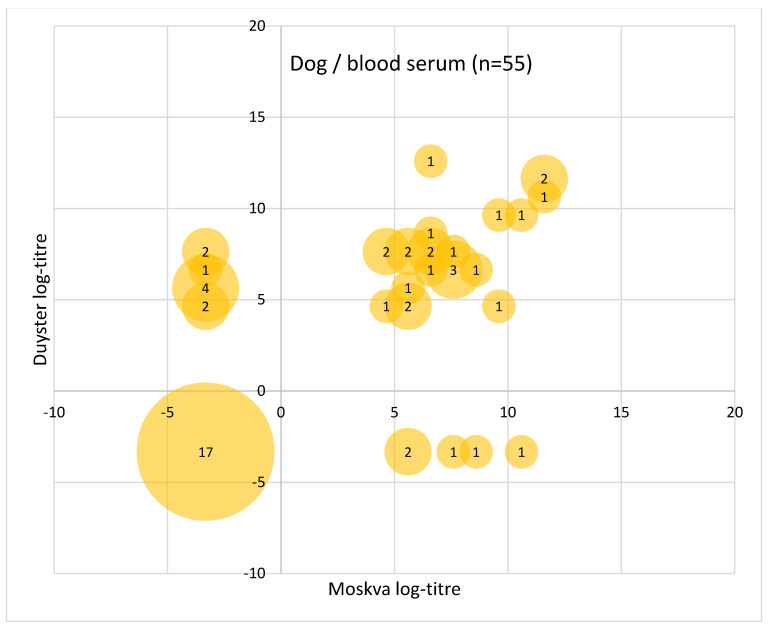
Plot of MAT test result pairs with both Grippotyphosa type strains Moskva (*x*-axis) and Duyster (*y*-axis) for the samples from dog. The log2 values of the MAT titres (log-titre) are shown as indicated in Table 1.

To better visualise the pairwise comparison of the test results with the two Grippotyphosa type strains for each sample, the log2 of the MAT titre levels (see Table 2) were plotted (see Figure 2, Figure 3 and Figure 4). Circles in the lower left quarter show consistent negative MAT-results (<100 for pigs and <25 for dogs and horses) with both strains. Circles in the upper right quarter show consistent positive results, where the diameter of the circles is proportional to the number of identical test results. The upper left quarter shows MAT test results that were positive only with Duyster and negative with Moskva, and the lower right quarter shows the reverse cases (Duyster negative, Moskva positive).

## 3. Results and Discussion

### 3.1. Differences in MAT Titres with Moskva and Duyster

In pigs, the final titre results in 92.6% (2774/2996) of the sera were completely consistent with the Moskva type strain as well as with the Duyster type strain (Table S1, see Figure 1a, difference in titre level = 0); in horses (Figure 1c) the final titre results were identical in 53.0% (61/115) of the samples, and in dogs (Figure 1b) only in 43.6% (24/55). In pigs, there is a more even distribution of differences in titres than in horses and dogs, with a slight shift in number of higher titres for Duyster compared to Moskva (see Figure 1a, difference in titre level < 0). In dogs (Figure 1b) and horses (Figure 1c), the deviations are more irregular, but here, too, a slight shift towards higher titres with Duyster compared to Moskva can be observed which could mean that the use of Duyster could be more sensitive for the detection of leptospiral infections by MAT. The number and proportion of concordant and discordant titre levels for all samples tested determined by using differences in titre levels are summarised in Figure 1 and Table S1.

### 3.2. MAT Titres by Animal Species and Sample Material by Grippotyphosa Type Strains Only

At least 92.5% of the tested sera (BS; n total: 2996) from pigs were negative for antibodies against the serovar Grippotyphosa. Using strain Duyster, 2.7% more pig sera (n = 81) were found to be positive with a titre of 100 or higher (Figure 2a).

In dogs, 47.3% of the sera (BS; n total: 55) were assessed as negative (titre < 25) by Grippotyphosa strain Moskva and 40.0% by Duyster. 34.6% of the dog sera had titres greater than or equal to 100 with Moskva and 40.0% with Duyster (Figure 2b).

In horses, 34.3% of the sera (BS; n total: 35) were assessed as negative (titre < 25) by strain Moskva and 31.4% by Duyster for Grippotyphosa. 48.6% of the horse sera had titres ≥ 100 with Moskva and 42.9% with Duyster (Figure 2c). The number of qualitative assessments (negative/positive) of the horse samples based on MAT titres was very consistent with both strains except for differences in two to five samples per sample type (Figure 2). The proportion of negatives (titre < 25) was significantly higher in AF samples (n total: 35) from the horses at around 88% than in VF and BS (Figure 2c). Among the VF samples, 48.9% (n = 22) were positive (titres ≥ 25) with Moskva, and 44.4% (n = 20) with Duyster. 51.1% (n = 23) of the VF samples from horses had titres ≥ 100 with Moskva and 55.6% (n = 25) with Duyster.

The results are consistent with the knowledge that when antibodies are detected in blood serum, it is a systemic infection of the host, whereas antibodies to leptospires in the VF reflect a local infection of the eye [15]. Only small amounts of the pathogen and/or antibodies against it are expected outside the vitreous body, e.g., in the aqueous fluid [41]. However, since the extraction of VF is more invasive than AF, AF is often tested for leptospires or antibodies against leptospires before vitrectomy [15]. It is important to note that the content of leptospires and anti-leptospiral antibodies in AF is always significantly lower, as can be observed here in addition to the results (Table S3), although the number of samples analysed by PCR and MAT was small. Reactivity with the two Grippotyphosa type strains is very similar but slightly more positive and higher in titres with Duyster compared to Moskva (Figure 1b and Table S1). Seropositivity for Grippotyphosa varies among animal species and among sample types (Figure 2).

A total of 91.6% of the pig sera showed no significant titres with Grippotyphosa and only 14 of 2996 porcine samples showed the highest titre with Grippotyphosa only. Thus, the serovar Grippotyphosa is generally of little importance for the pig (Figure S1), which has also been proven in various other studies [18,19,20]. Dogs and horses, however, show a different picture; in dogs, Grippotyphosa (20.0%) is the second most common serovar after Icterohaemorrhagiae (27.3%) by MAT, and in horses Grippotyphosa is the most frequently detected serovar (35.7%) in this study and in others [24,25,26,30,34,35,42].

In AF of horses, antibodies against serovar Grippotyphosa were detected much less frequently and also in lower amounts (lower titres), which was also observed in other studies [15,41]. At least 77.1% of AF samples were negative (titres < 25), whereas about 60% of the tested VF showed antibodies against serovar Grippotyphosa and titres up to 3200 and higher could be detected. In AF samples, leptospiral antibodies could only rarely be detected indirectly by MAT and even directly by PCR (2/35), 24 of 35 AF samples were both MAT-negative (for all serovars tested) and PCR-negative for leptospires (Table S3), but the number of samples tested with both methods was small. Similar results were also observed in other studies [43,44]. At least 42.9% of horse sera had significant levels of antibody (titres < 100) to Grippotyphosa, indicating systemic infection of horses with leptospires (Figure 2c) [12,27,45].

While the vast majority (91.6%) of the pig sera scored concordantly negative for serovar Grippotyphosa with both strains (Figure 3a), of the remaining 253 sera (Figure 3b), 116 were scored concordantly positive (3.9%) with both strains with a titre ≥ 100, although the titres determined per strain were not identical in every case. Only 28 pig sera were scored positive using serovar Grippotyphosa type strain Moskva but negative using strain Duyster (only 0.9% of all porcine sera), while 109 sera were scored positive using Duyster only and negative using Moskva (3.6%). In the vast majority of pig sera (95.6%), at least qualitatively uniform results could be obtained with both type strains with regard to serovar Grippotyphosa, i.e., irrespective of which strain was used for the MAT.

In the dogs, consistent with both type strains, 30.9% of sera were negative (17/55) and 43.6% were qualitatively positive (24/55) for serovar Grippotyphosa but not identical in the titres obtained per test strain (Figure 4). However, this means that overall at least 74.5% of the dog sera received a qualitatively consistent result regarding serovar Grippotyphosa. Five dog sera tested positive using only type strain Moskva and nine tested positive using only type strain Duyster. Unfortunately, it is not possible to clarify in this study which results of these samples are the true results. A follow-up study of Grippotyphosa-infected dogs, sufficient in number, with blood and urine samples and more comprehensive information from the medical records would be necessary to assess the diagnostic difference with both strains in dog.

**Figure 5:**
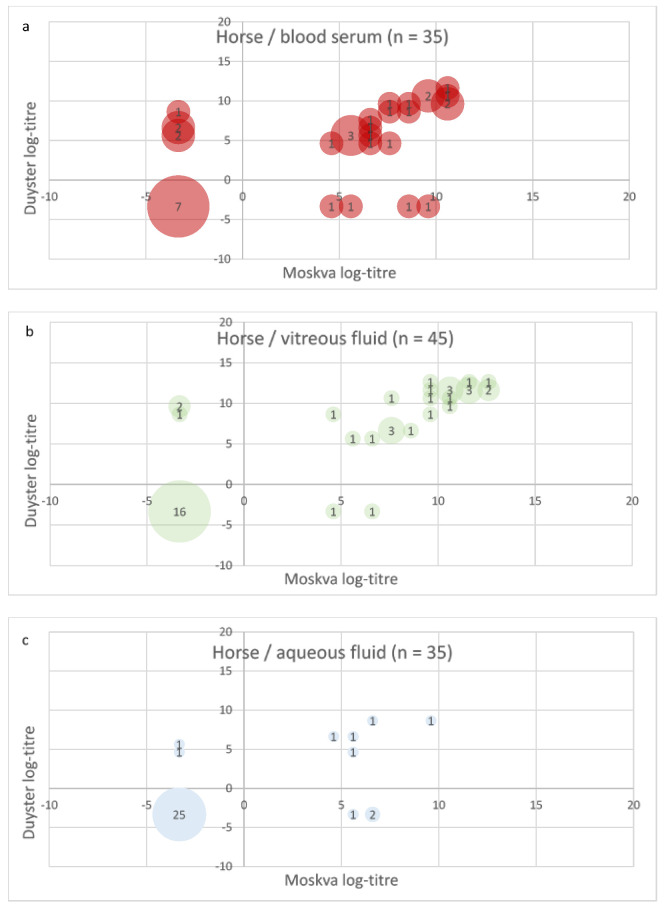
Plot of MAT test result pairs with both Grippotyphosa type strains Moskva and Duyster for the samples from horse; (**a**) blood serum (BS); (**b**) vitreous fluid (VF); (**c**) aqueous fluid (AF). The log2 values of the MAT titres (log-titre) are shown.

The results in the horse samples (Figure 5) show that at least 74.3% of the blood samples, 88.9% of the VF and 85.7% of the AF yielded a consistent result with both type strains, even if the titre levels were not identical, which could of course influence the evaluation of the result. As in the dog, follow-up studies of serovar Grippotyphosa-infected horses with more information on the animal and more samples would be useful for further direct and indirect detection methods for leptospires. In addition, ELISA results for the more sensitive general detection of a leptospiral infection should certainly be determined and considered in the future.

### 3.3. Assessment of Discrepant MAT Results with the Serovar Grippotyphosa Type Strains Considering Further Information and Supplementary Test Results

In order to be able to assess the contradictory results with the two strains, further results on these samples were consulted if they were available.

In the porcine sera (Table S4), there were only 19 sera that reacted with Grippotyphosa (Moskva) with the highest titre. Six of these were also identical in titre with the two strains. There were no porcine sera showing highest titre with Duyster and higher titres than with Moskva. There were five sera that were found positive only by Moskva, while no reaction could be detected with Duyster. In three of these sera, infection with leptospires is very likely because reactions with other Leptospira serovars were also detected in MAT. In the case of porcine sera, it makes little difference which strain is used for the examination by MAT. There were only five out of 2996 sera (0.16%) that would have been assessed as false negative if only Duyster had been used. Of course, this is also due to the fact that Grippotyphosa is only rarely responsible for an infection in pigs (Table S1 and Figure S1), which has been proven in many studies, even recent ones [18,19,20]. In conclusion, the results therefore show that the selection of one of the Grippotyphosa type strains has little impact in the context of the diagnosis of leptospiral infections in pigs using MAT.

A quarter of the dog sera were not evaluated consistently with the two serovar Grippotyphosa type strains (Figure 4). Paired urine samples were also available from 23 dogs, which could be tested for leptospires by PCR (Table S2). However, only in one of the urine samples could leptospires be detected by PCR. The serum of this dog was positive with Duyster but not with Moskva, which may support the already observed slightly more sensitive serological detection of leptospiral infection with Duyster instead of Moskva for the dog. Nevertheless, the number of paired samples from the dog is too small for a conclusive evaluation of the results. It could be assumed that additional urine samples for direct detection of leptospirosis are sent for diagnostic testing especially when there is a strong acute clinical suspicion of leptospirosis. Unfortunately, direct detection of leptospires in urine is also much more likely only in the convalescent stage of leptospirosis, even if intermittent leptospiruria must also be considered in the further course of the infection and in interpretation of diagnostic results [46]. EDTA blood from three dogs was also sent for testing by PCR, one sample of which was PCR-positive for pathogenic leptospires. This means that the dog was in the leptospiraemia phase. This blood sample also reacted serologically positive with both strains. These secondary findings show very clearly that the limited detectability of leptospirosis, especially due to the biphasic nature of the infection, makes it urgently necessary to use all available samples and methods, including clinical examination, for a meaningful diagnosis [14,46]. Otherwise, only small pieces of a mosaic are obtained as results with which a clear diagnostic picture cannot be achieved. Looking only at the discrepant results between Duyster and Moskva in the dog, these extreme differences in the results emerge in the vast majority of cases when higher titres are found with other serovars, suggesting that most of the divergent MAT results are when other serogroups, rather than Grippotyphosa, are likely to cause infection with Leptospira. (Table S2). There was only one case, which was negative with all tested serovars including strain Moskva but weakly positive with Duyster. No further sample or information was available for this dog. Nevertheless, the results show that for a clear evaluation of the two strains in the diagnostic use in MAT, a Grippotyphosa case–control study with a sufficient number of dogs seems to be necessary. However, this is not easy to realise, considering that many cases are often defined by MAT titres only [47] and there is also limited access to suitable cases [14,48].

In AF that tested negative by PCR for leptospires, one sample tested positive only with Duyster and another shows the highest titre with Copenhageni, but no titre (<25) with Moskva. In two of four VF samples that tested PCR positive, Duyster but not Moskva reacted positive. However, in a possible Grippotyphosa case, only Moskva and not Duyster reacted. In conclusion, there are few strongly divergent results between the two strains, but these also support the recommendation not to base the diagnosis of leptospirosis on a single test result. In the horse sera, in many cases only Moskva reacted, even if it was the highest titres in addition to cross reactions with other serovars. In contrast, Duyster and not Moskva reacted when higher titres with other serovars were detected.

Considering all results, Duyster may be slightly more sensitive when used in MAT for the horse, but the antibody response does not always appear to be Grippotyphosa-specific but Leptospira-specific. Unfortunately, even the PCR results of the animals with discrepant results between the two type strains could not help conclusively to decide whether the result with one or the other strain is the true result. However, this is also due to the fact that the detectability of leptospires directly and indirectly, based on antibodies against leptospires, is possible at different times during the course of infection, and there is only a limited phase in which the pathogen and antibodies against it can be detected simultaneously [1]. This is also shown by the comparison of PCR- and MAT-results of VF- and AF-samples from horse in a 4-panel board (Table S3) and in other studies [15]. Almost 50% of the VF samples were both PCR-positive and MAT-positive, although almost one-third of the samples were also PCR-negative but MAT-positive. Indeed, according to the literature, it must also be assumed that leptospires can initially be directly detected in the course of infection. Then antibodies, initially IgM and later IgG and other Igs are formed, and in the further course leptospires are no longer directly detectable in the local site of infection (here vitreous), but the antibodies remain detectable for much longer [1,11].

Therefore, in general a combination of direct and indirect pathogen detection remains the best recommendation for detecting infection with leptospires whenever possible [1]. Presumably, serum pairs or even follow-up studies of Grippotyphosa infection cases could help to further assess the suitability of the two serovar Grippotyphosa type strains in diagnostics. Unfortunately, serum pairs are rarely made available for routine diagnostics. However, especially in the animal species, e.g., horse and dog, as shown here and in other studies, where Grippotyphosa is a relevant serogroup in leptospiral infections, serum pairs should be obtained and examined for a more reliable diagnosis. Even if the PCR result is negative due to the nature of leptospirosis, an acute leptospiral infection can be detected more reliably by a serum pair instead of single serum samples.

## 4. Conclusions

Overall, however, there are few extremely discrepant results between the two serovar Grippotyphosa type strains. The results of this study indicate that strain Moskva seems to be more suitable for testing pig sera in cases of rare infections with Grippotyphosa compared to strain Duyster. In dogs and horses, where Grippotyphosa infections are more common, there are clearer differences between the two strains, with Duyster appearing to be more sensitive to the general detection of leptospiral infection but may be not Grippotyphosa-specific. However, these indications must definitely be assessed by further analyses of sufficient and secured leptospirosis cases in dogs and horses caused by Grippotyphosa, which will not be easy to find. Nevertheless, this comparative study of two Leptospira type stains has shown what further studies need to look like in order to assess the suitability of these two strains in diagnostics, especially for the dog and the horse.

## Figures and Tables

**Table 1:** Number and percentage of samples from horse, dog and pig tested positive with MAT for antibodies against *Leptospira* by serogroup.

Serogroup	Positive Samples per Serogroup *					
	Pig		Dog		Horse	
	Number	Percentage	Number	Percentage	Number	Percentage
Australis	437	14.6	9	16.4	14	12.2
Autumnalis	127	4.2	0	0.0	3	2.6
Canicola	11	0.4	0	0.0	1	0.9
Grippotyphosa	19	0.6	11	20.0	41	35.7
Ictero-haemorrhagiae	274	9.1	15	27.3	5	4.3
Pomona	42	1.4	3	5.5	4	3.5
Sejroe	13	0.4	2	3.6	3	2.6
Tarassovi	67	2.2	0	0.0	1	0.9
Multiple	185	6.2	10	18.2	9	7.8
Negative	1821	60.8	5	9.1	34	29.6
Total	2996	100.0	55	100.0	115	100.0

* with the highest titre for none (negative), one or more (multiple) serogroups.

**Table 2:** Titre, titre levels and log-titre for samples from: (a) horse and dog (companion animals); and from (b) pig (farm animals) for further analyses; * instead of <25 or <100, the log2 of 0.1 was calculated to obtain a value in the negative quadrant, and instead of >3200, the log2 of 6400 was calculated.

Titre (a)	<25 *	25	50	100	200	400	800	1600	3200	>3200 *
Titre level (a)	0 (negative)	1	2	3	4	5	6	7	8	9
Log2-titre (a)	−3.3	4.6	5.6	6.6	7.6	8.6	9.6	10.6	11.6	12.6
**Titre (b)**			**<100**	**100**	**200**	**400**	**800**	**1600**	**3200**	**>3200**
Titre level (b)			0 (negative)	3	4	5	6	7	8	9
Log2-titre (b)			−3.3	6.6	7.6	8.6	9.6	10.6	11.6	12.6

## Data Availability

The data that support the findings of this study are available from the corresponding author on request. Important data was provided as Supplementary Materials.

## Supplementary Materials

The following supporting information can be downloaded at: https://www.mdpi.com/article/10.3390/vetsci9090464/s1, Figure S1: Percentage of samples from (a) pig, (b) dog, and (c) horse tested by MAT for antibodies against Leptospira with the highest titre for none (Negative), one or more serogroups (Multiple); Table S1: Number and percentages of samples with differences in final titre levels of MAT between Grippotyphosa type strains Moskva and Duyster; Table S2: MAT results from the dog; Table S3: MAT- and PCR-results from the horse; Table S4: All available results from discrepant samples from pig; Table S5: All available results from discrepant samples from the dog; Table S6: All available results from discrepant samples from the horse.

## References

[1] Sykes J.E., Reagan K.L., Nally J.E., Galloway R.L., Haake D.A. (2022). Role of Diagnostics in Epidemiology, Management, Surveillance, and Control of Leptospirosis. Pathogens.

[2] OIE (2022). Leptospirosis. Manual of Diagnostic Tests and Vaccines for Terrestrial Animals.

[3] Guedes I.B., de Souza G.O., Castro J.F.P., Cavalini M.B., de Souza Filho A.F., Heinemann M.B. (2021). Usefulness of the Ranking Technique in the Microscopic Agglutination Test (MAT) to Predict the Most Likely Infecting Serogroup of Leptospira. Front. Vet. Sci..

[4] Wilkinson D.A., Edwards M., Benschop J., Nisa S. (2021). Identification of pathogenic *Leptospira* species and serovars in New Zealand using metabarcoding. PLoS ONE.

[5] Ahmed A., Engelberts M.F., Boer K.R., Ahmed N., Hartskeerl R.A. (2009). Development and validation of a real-time PCR for detection of pathogenic leptospira species in clinical materials. PLoS ONE.

[6] Stoddard R.A. (2013). Detection of pathogenic *Leptospira* spp. through real-time PCR (qPCR) targeting the LipL32 gene. Methods Mol. Biol..

[7] Ferreira A.S., Costa P., Rocha T., Amaro A., Vieira M.L., Ahmed A., Thompson G., Hartskeerl R.A., Inacio J. (2014). Direct detection and differentiation of pathogenic *Leptospira* species using a multi-gene targeted real time PCR approach. PLoS ONE.

[8] Miotto B.A., da Hora A.S., Taniwaki S.A., Brandao P.E., Heinemann M.B., Hagiwara M.K. (2018). Development and validation of a modified TaqMan based real-time PCR assay targeting the lipl32 gene for detection of pathogenic Leptospira in canine urine samples. Braz. J. Microbiol..

[9] Ahmed A.A., Goris M.G.A., Meijer M.C. (2020). Development of lipL32 real-time PCR combined with an internal and extraction control for pathogenic Leptospira detection. PLoS ONE.

[10] Perez L.J., Lanka S., DeShambo V.J., Fredrickson R.L., Maddox C.W. (2020). A Validated Multiplex Real-Time PCR Assay for the Diagnosis of Infectious *Leptospira* spp.: A Novel Assay for the Detection and Differentiation of Strains From Both Pathogenic Groups I and II. Front. Microbiol..

[11] Levett P.N. (2001). Leptospirosis. Clin. Microbiol. Rev..

[12] Ellis W.A. (2015). Animal leptospirosis. Curr. Top. Microbiol. Immunol..

[13] Goris M.G.A., Leeflang M.M.G. (2012). Establishment of Valid Laboratory Case Definition for Human Leptospirosis. J. Bacteriol. Parasitol..

[14] Miotto B.A., Tozzi B.F., Penteado M.S., Guilloux A.G.A., Moreno L.Z., Heinemann M.B., Moreno A.M., Lilenbaum W., Hagiwara M.K. (2018). Diagnosis of acute canine leptospirosis using multiple laboratory tests and characterization of the isolated strains. BMC Vet. Res..

[15] Wollanke B., Gerhards H., Ackermann K. (2022). Infectious Uveitis in Horses and New Insights in Its Leptospiral Biofilm-Related Pathogenesis. Microorganisms.

[16] Desai S., van Treeck U., Lierz M., Espelage W., Zota L., Sarbu A., Czerwinski M., Sadkowska-Todys M., Avdicova M., Reetz J. (2009). Resurgence of field fever in a temperate country: An epidemic of leptospirosis among seasonal strawberry harvesters in Germany in 2007. Clin. Infect. Dis..

[17] Nau L.H., Emirhar D., Obiegala A., Mylius M., Runge M., Jacob J., Bier N., Nockler K., Imholt C., Below D. (2019). Leptospirosis in Germany: Current knowledge on pathogen species, reservoir hosts, and disease in humans and animals. Bundesgesundheitsblatt Gesundh. Gesundh..

[18] Strutzberg-Minder K., Tschentscher A., Beyerbach M., Homuth M., Kreienbrock L. (2018). Passive surveillance of Leptospira infection in swine in Germany. Porcine Health Manag..

[19] Naudet J., Crespin L., Cappelle J., Kodjo A., Ayral F. (2022). Circulating serogroups of Leptospira in swine from a 7-year study in France (2011–2017). Porcine Health Manag.

[20] Macaluso G., Torina A., Blanda V., Guercio A., Lastra A., Giacchino I., D’Agostino R., Sciacca C., D’Incau M., Bertasio C. (2022). Leptospira in Slaughtered Fattening Pigs in Southern Italy: Serological Survey and Molecular Typing. Animals.

[21] Geisen V. (2009). Leptospirose bei Hunden in Süddeutschland.

[22] Gerlach T., Stephan I. (2007). Epidemiologische Situation der kaninen Leptospirose in Norddeutschland in den Jahren 2003–2006. Tierarztl Prax Ausg K Kleintiere Heimtiere.

[23] Mayer-Scholl A., Luge E., Draeger A., Nockler K., Kohn B. (2013). Distribution of Leptospira serogroups in dogs from Berlin, Germany. Vector Borne Zoonotic Dis..

[24] Delaude A., Rodriguez-Campos S., Dreyfus A., Counotte M.J., Francey T., Schweighauser A., Lettry S., Schuller S. (2017). Canine leptospirosis in Switzerland-A prospective cross-sectional study examining seroprevalence, risk factors and urinary shedding of pathogenic leptospires. Prev. Vet. Med..

[25] López M.C., Vila A., Rodón J., Roura X. (2019). *Leptospira* seroprevalence in owned dogs from Spain. Heliyon.

[26] Piredda I., Ponti M.N., Piras A., Palmas B., Pintore P., Pedditzi A., Chisu V. (2021). New Insights on Leptospira Infections in a Canine Population from North Sardinia, Italy: A Sero-Epidemiological Study. Biology.

[27] Ellis W.A. (2015). Horses and Donkeys. Leptospira and Leptospirosis.

[28] Divers T.J., Chang Y.F., Irby N.L., Smith J.L., Carter C.N. (2019). Leptospirosis: An important infectious disease in North American horses. Equine Vet. J..

[29] Hartskeerl R.A., Goris M.G., Brem S., Meyer P., Kopp H., Gerhards H., Wollanke B. (2004). Classification of leptospira from the eyes of horses suffering from recurrent uveitis. J. Vet. Med. B Infect. Dis. Vet. Public Health.

[30] Pikalo J., Sattler T., Eichinger M., Loitsch A., Sun H., Schmoll F., Schusser G.F. (2016). Occurrance of antibodies against *Leptospira* in horses in Middle Germany. Berl. Munch Tierarztl. Wochenschr..

[31] Wollanke B., Gerhards H., Brem S., Meyer P., Kopp H. (2004). Etiology of equine recurrent uveitis (ERU): Autoimmune disease or intraocular leptospiral infection?. Pferdeheilkunde.

[32] Loibl J., Gerhards H., Brem S., Wollanke B. (2018). Improving the laboratory diagnosis of leptospiral uveitis in horses by using an indirect ELISA for the detection of antibodies against *Leptospira* spp. in intraocular samples. Pferdeheilkunde Equine Med..

[33] Wollanke B., Geiger T., Gerhards H. (2018). Evaluation of “SNAP^®^ Lepto”-ELISA and comparison with MAT and PCR results for diagnosis of leptospiral uveitis in horses using intraocular samples. Pferdeheilkunde Equine Med..

[34] Dorrego-Keiter E., Tóth J., Dikker L., Sielhorst J., Schusser G.F. (2016). Detection of leptospira by culture of vitreous humor and detection of antibodies against leptospira in vitreous humor and serum of 225 horses with equine recurrent uveitis. Berl. Munch Tierarztl. Wochenschr..

[35] Geiger T., Gerhards H., Wollanke B. (2021). Detection of Anti-LipL32 Antibodies in Serum Samples from Horses with Chronic Intraocular Infection with *Leptospira* spp.. Pathogens.

[36] Fingerhut L., Yücel L., Strutzberg-Minder K., von Köckritz-Blickwede M., Ohnesorge B., de Buhr N. (2022). Ex Vivo and In Vitro Analysis Identify a Detrimental Impact of Neutrophil Extracellular Traps on Eye Structures in Equine Recurrent Uveitis. Front. Immunol..

[37] Wolff J.W., Bohlander H. (1952). Bovine leptospirosis; a survey of the epidemiology and serology and an investigation on the possible occurrence in bovines in the Netherlands. Doc. Med. Geogr. Trop..

[38] Steinen A.C., Schuurman J.L., Gravekamp C., Korver H., Terpstra W.J. (1992). Muskrats as carriers of pathogenic leptospires in The Netherlands. Antonie Van Leeuwenhoek.

[39] Levett P.N., Smythe L. (2008). International Committee on Systematics of Prokaryotes; Subcommittee on the taxonomy of Leptospiraceae: Minutes of the closed meeting, 18 September 2007, Quito, Ecuador. Int. J. Syst. Evol. Microbiol..

[40] OIE (2018). Leptospirosis. Manual of Diagnostic Tests and Vaccines for Terrestrial Animals.

[41] Malalana F., Blundell R.J., Pinchbeck G.L., McGowan C.M. (2017). The role of *Leptospira* spp. in horses affected with recurrent uveitis in the UK. Equine Vet. J..

[42] Mayer-Scholl A., Draeger A., Luge E., Ulrich R., Nockler K. (2011). Comparison of two PCR systems for the rapid detection of *Leptospira* spp. from kidney tissue. Curr. Microbiol..

[43] Faber N.A., Crawford M., LeFebvre R.B., Buyukmihci N.C., Madigan J.E., Willits N.H. (2000). Detection of *Leptospira* spp. in the aqueous humor of horses with naturally acquired recurrent uveitis. J. Clin. Microbiol..

[44] Sauvage A., Monclin S., Elansary M., Hansen P., Grauwels M. (2018). Detection of intraocular *Leptospira* spp. by real-time polymerase chain reaction in horses with recurrent uveitis in Belgium. Equine Vet. J..

[45] Haake D.A., Levett P.N. (2015). Leptospirosis in humans. Curr. Top. Microbiol. Immunol..

[46] Reagan K.L., Sykes J.E. (2019). Diagnosis of Canine Leptospirosis. Vet. Clin. N. Am. Small Anim. Pract..

[47] Ghneim G., Viers J., Chomel B., Kass P., Descollonges D., Johnson M. (2007). Use of a case-control study and geographic information system to determine environmental and demographic risk factors for canine leptospirosis. Vet. Res..

[48] Hetrick K., Harkin K.R., Peddireddi L., Henningson J. (2022). Evaluation by polymerase chain reaction assay of persistent shedding of pathogenic leptospires in the urine of dogs with leptospirosis. J. Vet. Intern. Med..

