# Supplementary material for: Comparison of Two Leptospira Type Strains of Serovar Grippotyphosa in Microscopic Agglutination Test (MAT) Diagnostics for the Detection of Infections with Leptospires in Horses, Dogs and Pigs
Source: Vet Sci. 2022 Aug 29;9(9):464. doi: 10.3390/vetsci9090464 (PMC9503138; doi:10.3390/vetsci9090464)
Supplement: Supplementary file 1 [file vetsci-09-00464-s001.zip › Figure S1 Percentage of samples.pdf]

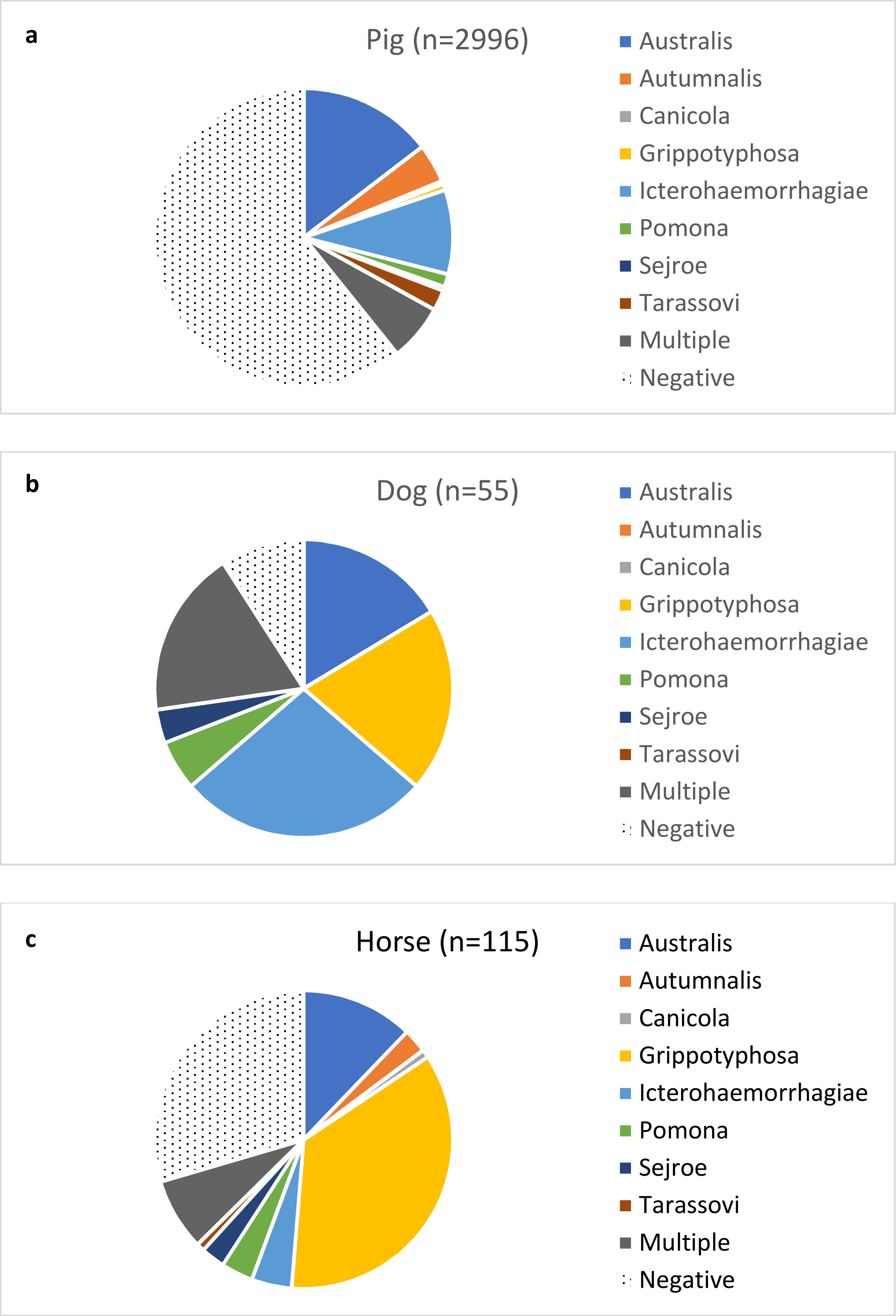

Figure S1. Percentage of samples from a) pig, b) dog, and c) horse tested by MAT for antibodies against Leptospira with the highest titre for none (Negative), one or more serogroups (Multiple).
