# Supplementary material for: Comparison of Two Leptospira Type Strains of Serovar Grippotyphosa in Microscopic Agglutination Test (MAT) Diagnostics for the Detection of Infections with Leptospires in Horses, Dogs and Pigs
Source: Vet Sci. 2022 Aug 29;9(9):464. doi: 10.3390/vetsci9090464 (PMC9503138; doi:10.3390/vetsci9090464)
Supplement: Supplementary file 1 [file vetsci-09-00464-s001.zip › Table S1.pdf]

**Table S1.** Number and percentages of samples with differences in final titre levels of MAT between Grippotyphosa type strains Moskva and Duyster.

| **Difference in final titre level** (<0: higher titre with Duyster 0: equal titre >0 higher titre with Moskva) | **Pig** | | **Dog** | | **Horse** | |
|---|---|---|---|---|---|---|
| | **Number of samples** | **Percentage** | **Number of samples** | **Percentage** | **Number of samples** | **Percentage** |
| **7** | 1 | 0.0 | 1 | 1.8 | 0 | 0.0 |
| **6** | 1 | 0.0 | 0 | 0.0 | 1 | 0.6 |
| **5** | 2 | 0.1 | 2 | 3.6 | 1 | 0.0 |
| **4** | 4 | 0.1 | 1 | 1.8 | 0 | 0.0 |
| **3** | 4 | 0.1 | 0 | 0.0 | 4 | 3.5 |
| **2** | 6 | 0.2 | 3 | 5.5 | 4 | 3.5 |
| **1** | 24 | 0.8 | 7 | 12.7 | 15 | 13.0 |
| **0** | 2774 | 92.6 | 24 | 43.6 | 61 | 53.0 |
| **-1** | 78 | 2.6 | 4 | 7.3 | 13 | 11.3 |
| **-2** | 32 | 1.1 | 7 | 12.7 | 7 | 6.1 |
| **-3** | 23 | 0.8 | 3 | 5.5 | 4 | 3.5 |
| **-4** | 20 | 0.7 | 2 | 3.6 | 1 | 0.9 |
| **-5** | 15 | 0.5 | 0 | 0.0 | 2 | 1.7 |
| **-6** | 6 | 0.3 | 1 | 1.8 | 2 | 1.7 |
| **-7** | 3 | 0.1 | 0 | 0.0 | 0 | 0.0 |
| Total | 2996 | 100.0 | 55 | 100.0 | 115 | 100.0 |
