# Supplementary material for: Comparison of Two Leptospira Type Strains of Serovar Grippotyphosa in Microscopic Agglutination Test (MAT) Diagnostics for the Detection of Infections with Leptospires in Horses, Dogs and Pigs
Source: Vet Sci. 2022 Aug 29;9(9):464. doi: 10.3390/vetsci9090464 (PMC9503138; doi:10.3390/vetsci9090464)
Supplement: Supplementary file 1 [file vetsci-09-00464-s001.zip › Table S2.pdf]

Table S2: MAT results from the dog

Dogs with paired urine samples	Duyster-positive	Duyster-negative (titre <25)	Total
Moskva-positive	8	1	9
Moskva-negative (titre <25)	5	9	14
Total	13	10	23

Dogs without paired urine samples	Duyster-positive	Duyster-negative (titre <25)	Total
Moskva-positive	16	4	20
Moskva-negative (titre <25)	4	8	12
Total	20	12	32
