# Supplementary material for: Comparison of Two Leptospira Type Strains of Serovar Grippotyphosa in Microscopic Agglutination Test (MAT) Diagnostics for the Detection of Infections with Leptospires in Horses, Dogs and Pigs
Source: Vet Sci. 2022 Aug 29;9(9):464. doi: 10.3390/vetsci9090464 (PMC9503138; doi:10.3390/vetsci9090464)
Supplement: Supplementary file 1 [file vetsci-09-00464-s001.zip › Table S3.pdf]

Table S3: MAT- and PCR-results from the horse

Horse-VF	PCR-positive	PCR-negative	Total
MAT-positive	22	14	36
MAT-negative (titre <25)	3	6	9
Total	24	20	45

Horse-AF	PCR-positive	PCR-negative	Total
MAT-positive	2	9	11
MAT-negative (<25)	0	24	24
Total	2	33	35
