# Supplementary material for: Comparison of Two Leptospira Type Strains of Serovar Grippotyphosa in Microscopic Agglutination Test (MAT) Diagnostics for the Detection of Infections with Leptospires in Horses, Dogs and Pigs
Source: Vet Sci. 2022 Aug 29;9(9):464. doi: 10.3390/vetsci9090464 (PMC9503138; doi:10.3390/vetsci9090464)
Supplement: Supplementary file 1 [file vetsci-09-00464-s001.zip › Table S4.pdf]

Table S4: All available results from discrepant samples from pig

Animal species	Sample type	Sample ID	Aus	Bra	Aut	Can	Cop	Ict	Pom	Har	Sax	Tar	Gri-Mos	Gri-Duy	Highest titre	with serovar/-group
pig	blood serum	20/18349-3	<100	<100	<100	<100	<100	<100	<100	<100	<100	<100	3200	1600	**3200**	**Gri**
pig	blood serum	20/15058-2	800	800	100	1600	800	400	<100	<100	<100	100	3200	100	**3200**	**Gri**
pig	blood serum	20/14560-7	800	1600	<100	200	1600	800	<100	<100	<100	1600	3200	3200	**3200**	**Gri**
pig	blood serum	20/13191-1	200	400	<100	100	800	800	<100	<100	<100	400	3200	1600	**3200**	**Gri**
pig	blood serum	20/17207-2	200	200	200	100	400	400	<100	<100	<100	100	3200	<100	**3200**	**Gri**
pig	blood serum	20/12535-2	200	200				200					1600	800	**1600**	**Gri**
pig	blood serum	20/16262-8	400	200	<100	<100	100	100	400	<100	<100	100	800	400	**400**	**multiple**
pig	blood serum	20/14480-6	200	200	100	100	200	400	<100	<100	<100	400	800	800/3200	**3200**	**Gri**
pig	blood serum	20/14475-8	<100	100	<100	<100	<100	<100	<100	<100	<100	<100	800	800	**800**	**Gri**
pig	blood serum	20/14475-6	100	100	<100	<100	<100	<100	<100	<100	<100	<100	800	800	**800**	**Gri**
pig	blood serum	20/13088-5	100	100	<100	100	100	100	<100	<100	<100	100	800	800	**800**	**Gri**
pig	blood serum	20/17207-1	100	200	200	<100	100	100	<100	<100	<100	100	800	<100	**800**	**Gri**
pig	blood serum	20/14301-2	<100	<100	<100	<100	<100	<100	<100	<100	<100	<100	800	<100	**800**	**Gri**
pig	blood serum	20/18663-9	<100	100	<100	<100	200	100	<100	<100	<100	100	400	400	**400**	**Gri**
pig	blood serum	20/13408-5	<100	<100	<100	<100	<100	100	<100	<100	<100	<100	400	200	**400**	**Gri**
pig	blood serum	20/14886-2	<100	<100	<100	<100	<100	<100	<100	<100	<100	<100	100	100	**100**	**Gri**
pig	blood serum	20/14182-6	<100	<100	<100	<100	<100	<100	<100	<100	<100	<100	100	3200	**100/3200**	**Gri**
pig	blood serum	20/16975-8	<100	<100	<100	<100	<100	<100	<100	<100	<100	<100	100	<100	**100**	**Gri**
pig	blood serum	20/15795-7	<100	<100	<100	<100	<100	<100	<100	<100	<100	<100	100	<100	**100**	**Gri**

Samples retested

Animal species	Sample type	Sample ID	Aus	Bra	Aut	Can	Cop	Ict	Pom	Har	Sax	Tar	Gri-Mos	Gri-Duy	Highest titre	with serovar/-group
pig	blood serum	20/18349-3	<100	<100	<100	<100	<100	<100	<100	<100	<100	<100	3200	1600	**3200**	**Gri**
pig	blood serum	20/15058-2	400	800	100	1600	800	400	<100	<100	<100	100	3200	100	**3200**	**Gri**
pig	blood serum	20/14560-7	800	1600	<100	200	1600	800	<100	<100	<100	1600	3200	3200	**3200**	**Gri**
pig	blood serum	20/13191-1	200	400	<100	100	800	800	<100	<100	<100	200	3200	1600	**3200**	**Gri**
pig	blood serum	20/17207-2	200	200	200	100	400	400	<100	<100	<100	100	3200	<100	**3200**	**Gri**
pig	blood serum	20/12535-2	200	200				200					1600	800	**1600**	**Gri**
pig	blood serum	20/16262-8	200	200	<100	<100	100	100	400	<100	<100	100	800	400	**400**	**multiple**
pig	blood serum	20/14480-6	200	200	100	100	400	400	<100	<100	<100	400	800	800/3200	**3200**	**Gri**
pig	blood serum	20/14475-8	<100	100	<100	<100	<100	<100	<100	<100	<100	<100	800	800	**800**	**Gri**
pig	blood serum	20/14475-6	100	100	<100	<100	<100	<100	<100	<100	<100	<100	800	800	**800**	**Gri**
pig	blood serum	20/13088-5	na	na	na	na	na	na	na	na	na	na	na	na		
pig	blood serum	20/17207-1	100	200	200	<100	100	100	<100	<100	<100	100	800	<100	**800**	**Gri**
pig	blood serum	20/14301-2	<100	<100	<100	<100	<100	<100	<100	<100	<100	<100	800	<100	**800**	**Gri**
pig	blood serum	20/18663-9	<100	100	<100	<100	200	100	<100	<100	<100	100	400	400	**400**	**Gri**
pig	blood serum	20/13408-5	<100	<100	<100	<100	<100	100	<100	<100	<100	<100	400	200	**400**	**Gri**
pig	blood serum	20/14886-2	<100	<100	<100	<100	<100	<100	<100	<100	<100	<100	100	100	**100**	**Gri**
pig	blood serum	20/14182-6	<100	<100	<100	<100	<100	<100	<100	<100	<100	<100	200	3200	**200/3200**	**Gri**
pig	blood serum	20/16975-8	<100	<100	<100	<100	<100	<100	<100	<100	<100	<100	100	<100	**100**	**Gri**
pig	blood serum	20/15795-7	<100	<100	<100	<100	<100	<100	<100	<100	<100	<100	100	<100	**100**	**Gri**

na: no sample available for a repeated testing
