# Supplementary material for: Comparison of Two Leptospira Type Strains of Serovar Grippotyphosa in Microscopic Agglutination Test (MAT) Diagnostics for the Detection of Infections with Leptospires in Horses, Dogs and Pigs
Source: Vet Sci. 2022 Aug 29;9(9):464. doi: 10.3390/vetsci9090464 (PMC9503138; doi:10.3390/vetsci9090464)
Supplement: Supplementary file 1 [file vetsci-09-00464-s001.zip › Table S5.pdf]

Table S5: All available results from discrepant samples from the dog

Animal species	Sample type	Sample ID	Aus	Bra	Aut	Can	Cop	Ict	Pom	Har	Sax	Tar	Gri-Mos	Gri-Duy	Highest titre	with serovar/-group	Lepto-PCR paired urine (ct)
dog	blood serum	20/11755-1	800	1600	100	<25	200	400	200	<25	3200	<25	200	<25	**3200**	**Sax**	negative
		20/11755-1	800	1600	100	<25	200	200	200	<25	3200	<25	200	<25	**3200**	**Sax**	
dog	blood serum	20/14737-1	400	3200	400	50	800	200	1600	<25	<25	100	1600	<25	**3200**	**Bra**	X
		20/14737-1	800	3200	400	25	800	200	1600	<25	<25	100	1600	<25	**3200**	**Bra**	
dog	blood serum	20/12060-1	<25	<25	<25	400	800	25	50	<25	<25	<25	400	<25	**800**	**Cop**	X
		20/12060-1	<25	<25	<25	400	800	25	50	<25	<25	<25	400	<25	**800**	**Cop**	
dog	blood serum	20/14421-1	50	200	<25	100	<25	<25	<25	<25	<25	<25	50	<25	**200**	**Bra**	X
		20/14421-1	100	200	<25	100	<25	<25	<25	<25	<25	<25	50	<25	**200**	**Bra**	
dog	blood serum	20/14864-1	<25	100	<25	<25	100	50	<25	<25	<25	<25	50	<25	**100**	**multiple**	X
dog	blood serum	20/12794-1	<25	<25	<25	<25	<25	25	<25	<25	<25	<25	<25	25	**25**	**Ict**	negative
dog	blood serum	20/13012-1	<25	<25	<25	<25	<25	50	50	<25	<25	<25	<25	25	**50**	**multiple**	**positive (40)**
dog	blood serum	20/15454-1	50	100	<25	100	200	100	<25	<25	<25	<25	<25	50	**200**	**Cop**	negative
		20/15454-1	50	100		100	200	200	<25	<25	<25	<25	<25	50	**200**	**ICT**	
dog	blood serum	20/15799-1	100	100	<25	<25	400	200	<25	<25	<25	<25	<25	200	**400**	**Cop**	X
		20/15799-1	100	100	<25	<25	400	200	<25	<25	<25	<25	<25	200	**400**	**Cop**	
dog	blood serum	20/16098-1	50	100	<25	50	50	100	25	<25	<25	<25	<25	100	**100**	**multiple**	negative
dog	blood serum	20/16627-1	<25	200	200	100	<25	<25	800	<25	<25	<25	<25	50	**800**	**Pom**	X
dog	blood serum	20/16792-1	100	200	<25	100	800	200	<25	<25	<25	<25	<25	200	**800**	**Cop**	X
		20/16792-1	100	200	<25	<25	400	200	<25	<25	<25	<25	<25	100	**400**	**Cop**	
dog	blood serum	20/16802-1	<25	<25	<25	<25	<25	<25	<25	<25	<25	<25	<25	50	**<25**	negative	X
		20/16802-1	<25	<25	<25	<25	50	<25	<25	<25	<25	<25	<25	50	**<25**	negative	
dog	blood serum	20/18226-1	50	100	<25	<25	50	50	<25	<25	<25	<25	<25	50	**100**	**Bra**	negative
		20/18226-1	50	100	<25	<25	50	25	<25	<25	<25	<25	<25	50	**100**	**Bra**	

X: no sample for PCR-testing available
