# Supplementary material for: Comparison of Two Leptospira Type Strains of Serovar Grippotyphosa in Microscopic Agglutination Test (MAT) Diagnostics for the Detection of Infections with Leptospires in Horses, Dogs and Pigs
Source: Vet Sci. 2022 Aug 29;9(9):464. doi: 10.3390/vetsci9090464 (PMC9503138; doi:10.3390/vetsci9090464)
Supplement: Supplementary file 1 [file vetsci-09-00464-s001.zip › Table S6.pdf]

**Table S6:** All available results from discrepant samples from the horse

| Animal species | Sample type | Sample ID | Aus | Bra | Aut | Can | Cop | Ict | Pom | Har | Sax | Tar | Gri-Mos | Gri-Duy | Highest titre | with serovar/-group | Lepto-PCR (ct) |
|---|---|---|---|---|---|---|---|---|---|---|---|---|---|---|---|---|---|
| horse | aqueous fluid | 20FE/163-6 | <25 | <25 | <25 | <25 | <25 | <25 | <25 | <25 | <25 | <25 | <25 | 25 | **<25** | negative | negative |
| | | 20FE/163-6 | <25 | <25 | <25 | <25 | <25 | <25 | <25 | <25 | <25 | <25 | <25 | <25 | **<25** | negative | |
| horse | aqueous fluid | 20FE/172-5 | <25 | <25 | <25 | <25 | <25 | <25 | <25 | <25 | <25 | <25 | 100 | <25 | **100** | **Gri** | negative |
| | | 20FE/172-5 | <25 | <25 | <25 | <25 | <25 | <25 | <25 | <25 | <25 | <25 | 50 | <25 | **50** | **Gri** | |
| horse | aqueous fluid | 20FE/178-1 | 100 | 200 | 50 | 50 | 800 | 400 | <25 | <25 | 50 | 25 | <25 | 50 | **800** | **Cop** | negative |
| | | 20FE/178-1 | 50 | 50 | <25 | 25 | 400 | 200 | <25 | <25 | 25 | <25 | <25 | 50 | **400** | **Cop** | |
| horse | aqueous fluid (L) | 20FE/187-6 | <25 | <25 | <25 | <25 | <25 | <25 | <25 | <25 | <25 | <25 | 50 | <25 | **50** | **Gri** | negative |
| | | 20FE/187-6 | <25 | <25 | <25 | <25 | <25 | <25 | <25 | <25 | <25 | <25 | 25 | <25 | **50** | **Gri** | |
| horse | aqueous fluid (R) | 20FE/187-5 | <25 | <25 | <25 | <25 | <25 | <25 | <25 | <25 | <25 | <25 | 100 | <25 | **100** | **Gri** | negative |
| horse | vitreous fluid | 20FE/161-1 | <25 | <25 | <25 | <25 | <25 | <25 | <25 | <25 | <25 | <25 | 25 | <25 | **25** | **Gri** | negative |
| | | 20FE/161-1 | <25 | <25 | <25 | <25 | <25 | <25 | <25 | <25 | <25 | <25 | 25 | <25 | **25** | **Gri** | |
| horse | vitreous fluid | 20FE/138-2 | <25 | <25 | <25 | <25 | <25 | 1600 | <25 | 25 | <25 | <25 | <25 | 800 | **1600** | **Ict** | **positive (33)** |
| | | 20FE/138-2 | <25 | <25 | <25 | <25 | <25 | 800 | <25 | 25 | <25 | <25 | <25 | 400 | **800** | **Ict** | |
| horse | vitreous fluid | 20/18594-1 | <25 | <25 | <25 | <25 | <25 | <25 | <25 | <25 | <25 | <25 | <25 | 800 | **<25** | negative | **positive (34)** |
| horse | vitreous fluid | 20/18224-1 | <25 | <25 | <25 | <25 | <25 | <25 | <25 | <25 | <25 | <25 | <25 | 400 | **<25** | negative | **positive (38)** |
| horse | vitreous fluid | 20FE/176-2 | <25 | <25 | <25 | 25 | <25 | <25 | <25 | <25 | <25 | <25 | 100 | <25 | **100** | **Gri** | **positive (38)** |
| | | 20FE/176-2 | <25 | <25 | <25 | 25 | <25 | <25 | <25 | <25 | <25 | <25 | 100 | <25 | **100** | **Gri** | |

same master number/colour = same horse

Continuation **Table 6**

| Animal species | Sample type | Sample ID | Aus | Bra | Aut | Can | Cop | Ict | Pom | Har | Sax | Tar | Gri-Mos | Gri-Duy | Highest titre | with serovar/-group | Lepto-PCR (ct) |
|---|---|---|---|---|---|---|---|---|---|---|---|---|---|---|---|---|---|
| horse | blood serum | 20FE/147-1 | 25 | 200 | 50 | 50 | <25 | <25 | <25 | <25 | 200 | 100 | <25 | 50 | **200** | **multiple** | X |
| horse | blood serum | 20FE/155-1 | <25 | 25 | 25 | 50 | 25 | 50 | <25 | 200 | 400 | 100 | 800 | <25 | **800** | **Gri** | X |
| horse | blood serum | 20FE/158-1 | 25 | 50 | 50 | <25 | <25 | <25 | <25 | <25 | <25 | 25 | 50 | <25 | **50** | **multiple** | X |
| | | 20FE/158-1 | 50 | 50 | 50 | <25 | <25 | 50 | <25 | <25 | <25 | 25 | 50 | <25 | **50** | **multiple** | |
| horse | blood serum | 20FE/162-1 | 50 | 50 | 25 | 25 | 50 | 50 | <25 | <25 | <25 | <25 | 400 | <25 | **400** | **Gri** | X |
| | | 20FE/162-1 | 25 | 25 | 25 | 25 | 50 | 50 | <25 | <25 | <25 | <25 | 800 | <25 | **800** | **Gri** | |
| horse | blood serum | 20FE/163-1 | 100 | 200 | <25 | 25 | <25 | <25 | <25 | <25 | <25 | <25 | <25 | 50 | **200** | **Bra** | X |
| | | 20FE/163-1 | 50 | 200 | <25 | 25 | <25 | <25 | <25 | <25 | <25 | <25 | <25 | 50 | **200** | **Bra** | |
| horse | blood serum | 20FE/167-1 | <25 | <25 | <25 | <25 | <25 | <25 | <25 | <25 | <25 | <25 | <25 | 400 | **<25** | negative | X |
| | | 20FE/167-1 | <25 | <25 | <25 | <25 | <25 | <25 | <25 | <25 | <25 | <25 | <25 | 200 | **<25** | negative | |
| horse | blood serum | 20FE/168-1 | <25 | <25 | <25 | 50 | <25 | <25 | <25 | <25 | <25 | <25 | <25 | 100 | **50** | **Can** | X |
| horse | blood serum | 20FE/173-1 | <25 | 100 | <25 | <25 | <25 | <25 | <25 | <25 | <25 | <25 | <25 | 100 | **100** | **Bra** | X |
| horse | blood serum | 20FE/185-1 | 25 | 50 | <25 | <25 | <25 | 25 | <25 | <25 | <25 | <25 | 25 | <25 | **50** | **Bra** | X |
| | | | 25 | 50 | <25 | <25 | <25 | 25 | <25 | <25 | <25 | <25 | 25 | <25 | **50** | **Bra** | |

same master number/colour = different samples from the same horse

X: examination not useful
